# Cultural beliefs, attitudes and perceptions of lactating mothers on exclusive breastfeeding in The Gambia: an ethnographic study

**DOI:** 10.1186/s12905-023-02163-z

**Published:** 2023-01-13

**Authors:** Sering A. L. Sosseh, Amadou Barrow, Zxyyann Jane Lu

**Affiliations:** 1 International Health Program, National Yang-Ming University, Hsinchu, Taiwan; 2 Department of Public and Environmental Health, School of Medicine & Allied Health Sciences, University of The Gambia, Kanifing, The Gambia; 3 Department of Bioscience Technology, Chung Yuan Christian University, Taoyüan, Taiwan

**Keywords:** Breastfeeding, Exclusive breastfeeding, Infant nutrition, Gambia, Cultural beliefs

## Abstract

**Background:**

WHO/UNICEF recommends that women in resource-poor developing countries- like the Gambia, should exclusively breastfeed their infants for the first six months of their lives because of its health benefits to both mother and infant. The study aimed to explore the cultural beliefs, attitudes, and perceptions of lactating mothers towards exclusive breastfeeding in The Gambia.

**Methods:**

This was a qualitative ethnographic study of culture-sharing groups of mothers with infants 4 to 6 months old. The study was conducted from July to October 2014 and data collection was done through a face-to-face, in-depth interview and moderate participant observation. The study recruited 22 breastfeeding mothers attending government health facilities in the Kanifing Municipality. The collected data were transcribed verbatim and analyzed through a constant comparison method generating six cultural themes, each with sub-themes.

**Results:**

Baby’s welfare is traditionally based on the types of food mother’s eat. To this end, mothers reportedly shunned eating green leafy vegetables, liquid and hot foods for their infants’ wellbeing. Encounters such as weight loss, nipple inflammation, and backache, which mothers associated with hyper latching and sitting for prolonged breastfeeding, respectively, were among major undesirable physical effects revealed by the participants. Furthermore, the necessity of giving water to infants for their survival was illustrated as a barricade to exclusive breastfeeding practices. Likewise, the entrenched practice of giving charm water to instill the Islamic faith and shielding infants against evil spirits was another factor influencing exclusive breastfeeding practices. Finally, the belief that breast milk adequacy is based on breast size and nurturing men’s physical strength by starting prelacteal feeds early in infancy also contributes to the meek exclusive breastfeeding rate among mothers.

**Conclusion:**

This study could be a gazette piece for effective policy making and enhance nurses’ cultural sensitivity while caring for lactating mothers. Cultural meanings of health care behaviors in lactating mothers challenge universally applying guidelines of exclusive breastfeeding to all societies. The study findings could benefit healthcare providers in informing policies and designing culturally adaptive and acceptable community-based breastfeeding intervention programs in resource-limited settings.

## Introduction

Exclusive breastfeeding is the feeding of newborns with only breast milk and no other food or liquid, not even water—except for oral rehydration solution, drops/syrups of vitamins, minerals or medicines, from birth to six months [1]. When exclusive breastfeeding is done in the first six months of life, it reduces infant morbidity and mortality [2]. Its effects on child survival, growth, and maternal health are well-documented [2, 3]. The natural and ideal way of feeding infants provides babies all the energy and nutrients they need for the first six months of their lives [1]. It also provides babies with nutritious and easily digestible food, especially the first thick yellow milk called colostrum. Besides providing warmth and strengthening the immune system, colostrum contains anti-bacterial and anti-viral agents with high quantities of vitamin A from the mother's body, which helps sick children recover [4]. It decreases the occurrence and severity of childhood diseases like diarrhea, respiratory or ear infections, minimizing infant morbidity and mortality [5, 6]. In a WHO research, breastfeeding was twice as protective as not breastfeeding in the first year of life [1].

Globally, exclusive breastfeeding is promoted as the 'best newborn feeding approach' [1]. Increasing scientific data shows that exclusive breastfeeding improves baby survival [3, 7–10]. It's one of the most effective ways to minimize newborn morbidity and death in resource-limited contexts, where improper nursing techniques induce child malnutrition, a primary cause of infant mortality [1]. Continued breastfeeding lowers dehydration, diarrhea intensity and duration, and malnutrition in newborns, reducing infant mortality. Exclusively breastfed infants are six times less likely to die from diarrhea or acute respiratory infections than those not [1, 11]. Exclusive breastfeeding, a hygienic source of energy, protein, fat, vitamins, and other nutrients for newborns optimizes physical growth and development [8, 10]. It also promotes frequent interaction between mothers and infants, which is vital for a baby's brain development [10]. Others have estimated that inadequate breastfeeding habits cause millions of child morbidity and mortality yearly [10]. If practiced as advised, optimal exclusive breastfeeding alone can save the lives of roughly 820,000 under 5 children annually worldwide, thus contributing to goal 3 of Sustainable Development Goal (SDG) [1].

Exclusive breastfeeding is good for babies and mothers. It lessens the incidence of postpartum hemorrhage, breast, and ovarian cancer [7]. It conserves mothers' iron levels and delays fertility return, reducing maternal anaemia and promoting child spacing [2]. Exclusive breastfeeding benefits the family, health care system, and employers socially and economically [12, 13]. Exclusively breastfed babies require fewer sick visits, medicines, and hospitalizations than non-exclusively breastfed babies, decreasing their health care needs [9, 14–16]. Exclusive breastfeeding is a naturally renewable and sustainable resource that requires no fuel for preparation, packaging, or disposal [1]. This protects the environment and saves the mother’s money that would have been spent on infant feeds and treating illnesses due to contaminated and inadequate breast milk substitutes [13].

Almost 98% of Gambia's children ever breastfed which includes mixed and exclusive while only 47% of infants under six months are exclusively breastfed, despite widespread awareness programs [17, 18]. The survey estimated the under-five and infant mortality rates to be 56/1000 and 42/1000 live births, respectively and these were higher than 2013 GDHS survey estimates [17, 19]. Several studies have been undertaken in The Gambia and other countries to understand exclusive breastfeeding [20–22]. While some have investigated the promotion and protection of breastfeeding [23, 24], importance of cultural beliefs, attitude and perceptions towards exclusive breastfeeding [25–27] the health outcomes of exclusive vs. non-exclusive breastfeeding [16, 28], and the protective effects of breastfeeding against infection [29] and others factors associated with non-exclusive breastfeeding [12, 28, 30].

However, little attempt has been made to investigate how cultural beliefs, attitudes and perceptions influence the practice of exclusive breastfeeding, especially in Sub-Saharan Africa, where indigenous cultural beliefs are entrenched. The study would provide additional insights in our attempt to understand the roles of cultural beliefs, attitudes and perceptions of maternal women towards the practice of exclusive breastfeeding in resource-limited settings. Given the significance of exclusive breastfeeding, especially towards attaining SDGs- goal 3 on achieving good health, this study attempts to identify the cultural beliefs, attitudes, and perceptions of lactating mothers towards exclusive breastfeeding and to describe the socio-cultural factors influencing the practice of exclusive breastfeeding among lactating mothers in the Gambia.

## Methods

The methodology used in this study was an ethnographic approach. This choice was made in line with the study aims and research questions. Its appropriateness was increased by the depth of data needed to comprehend and explain participants’ cultural beliefs, attitudes, and perceptions about exclusive breastfeeding practices. Ethnographic interview and moderate participant observation using a self-designed interview guide and observation checklist was used in the data collection. Depending on the participants’ choice, interviews were conducted in either the health center (RCH clinic) or the chosen environment (home or workplace).

Using the observation guide, few participants were also moderately observed in their natural settings/environment (markets, gardens, shops, homes, etc.) where they carried out their daily activities. These observations permitted researchers to immerse themselves into participants' daily activities and garner in-depth data. During the observation, the researchers took special care and attention to detail, context and difference in what the participants mentioned.

Naturalism was used to evaluate seen reality holistically and contextually. Meaning participants were monitored in their natural environment, noting who they interacted with and for how long. This gives the researchers first-hand information and understanding of social structures and activities and a thick description of participants’ behavior, language and words from the native point of view. Throughout researcher-participant interactions, the researchers consider their positions to avoid influencing the debate or observation. Before any formal interaction, researchers will introduce themselves, explain the study’s goal, and seek their involvement.

### Data collection process

Data collection in this study took the following approach: locating the study site and individuals, gaining access and making rapport, theoretical sampling, collecting data, recording information, resolving field issues and securing data storage. Detailed description of the data collection process is highlighted below.

### Study site and participants

Since ethnographic research attempts to study participants' cultural patterns and perspectives in their natural setting, understanding respondents’ language and culture is crucial. It reduces the possibility of mistranslating and/or misunderstanding the original and actual responses of the informants. Concerning this, Kanifing Municipality has been chosen as the study site, and the three key languages (Wollof, Mandinka and Fulla) were the communication medium during the interviews. It is about 13 km from the capital city of the Gambia with a total population of 382,096 (192, 417 males and 189, 679 females), representing about 24% of the country's total population. The estimated number of households in the municipality is about 67,119, with an annual growth rate of 1.7, making the average household size of 5.7 [31]. Furthermore, it has a total land surface area of 75.5 square kilometers and population density of 4,478 persons per square kilometer [32]. This municipality's common economic activities are urban agriculture, tie and dye, petty trading (including fruit vendors), and selling of forest products and seafood. The hotel and construction industries are also major sources of employment for the youths [32].

### Theoretical sampling

**Fig. 1:**
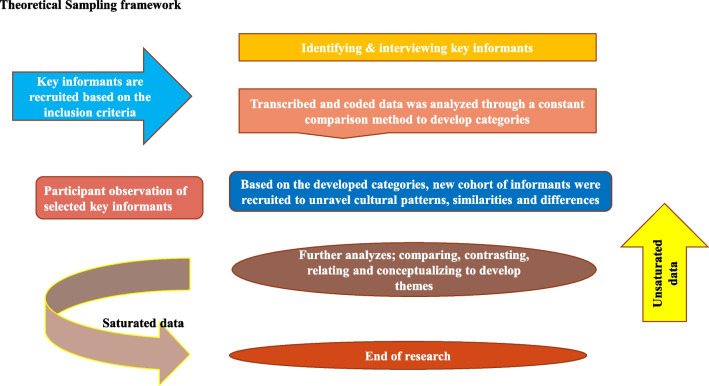
Theoretical Sampling framework

Theoretical sampling, as defined by Corbin 2008, “is a method of data collection that is based on concepts/themes derived from a data” [33]. This approach was utilized as a sampling strategy as it responds to information needs and is not predefined. Since the study attempts to unravel new notions but does not confirm theories, the researchers traveled to the field to collect data on them as shown in Fig. 1.

With this approach, obtained data was simultaneously gathered and processed to produce concepts/themes, replied to issues arising from the concepts, and, if necessary, returned to the field to collect more data to assure a comprehensive and holistic description. This procedure proceeded until data saturation, or until all concepts were finalized.

### Inclusion and exclusion criteria

*Inclusion criteria* Lactating mothers with infants aged 4–6 months attending public health facilities within the Kanifing Municipality.

*Exclusion criteria* Lactating mothers and/or infants with known chronic health conditions and single-parent mothers attending the same facilities.

### Data collection techniques

#### Key informants

Key informants of this study were breastfeeding mothers with infants aged 4–6 months. The first set of participants were identified and recruited at the RCH clinic of the specified health facility using screening and weighing tables. After identifying and recruiting informants on each clinic day, data were organized chronologically by date and time in an excel table. Those who chose to be interviewed in the institution were interviewed the same day and observed afterwards. Participants who requested to be questioned outside the health facility were visited at the arranged time and place. After reviewing the first group's data, the second group was picked at the researcher's option to answer follow-up questions.

#### Ethnographic interview and participant observation

This study used an ethnographic interview and moderate participant observation to collect data. From July to October 2014, consenting mothers were interviewed using an interview guide. Self-designed grand tour questions describe interviewees' cultural views and experiences. After evaluating descriptive data, follow-up questions were devised to uncover cultural patterns and domains and find similarities and contrasts.

Data was collected through an in-depth interview using the interview guide. Each participant was interviewed once for an hour. The researcher also records all interviews with a digital audio tape recorder. Using grand tour questions, a total of 36 interviews were conducted, of which, 17 were general discussions and the remaining 19 were follow-up discussions classifying issues with mothers. Each interview focused on mothers' exclusive breastfeeding experiences, beliefs and barriers, asking questions like reasons for exclusively/not exclusively breastfeeding, decision making about infant feeding, experiences, encounters and barriers to exclusive breastfeeding, socio-cultural and physical factors associated with exclusive breastfeeding, available support for breastfeeding, who is involved and what assistance do they give, thoughts about milk sufficiency, and feasibility. All interviews were conducted in the participant's original language (Wolof, Mandinka, and Fulla), and an interpreter was engaged when necessary. The researchers explains the study's goal to an eligible participant and seeks her agreement. The researchers discusses interview details with consenting volunteers outside the health center.

Participant observation was also used to observe participants' daily lives. This reveals what participants do, not what they say. It also helps researchers grasp what participants say and do holistically. With sensitivity to numerous realities and cultural relativism, it helps put interview information into context. In the first visit, researchers observed the informant's general environment, including what setting and activities she engages in, how much time she spends on them, whom she interacts with, family structure, relation and interactions, social networks and decision-making power, rapport with colleagues, support in domestic chores and infant feeding, and breastfeeding practices.

#### Information recording and data storage

Digital sound recorders were used to record interviews. Recorded information was transcribed verbatim, saved in a password-protected computer, and backed up daily. Depending on the activity, an interview or observation guide was utilized before, during, and after. All audio tapes, files, pictures, and field notes including interview and observation data were securely saved after data processing and presentation.

#### Data analysis

This study simultaneously collected and analyzed data utilizing the spiral circle approach, which included managing data, reading and selective coding, describing, classifying, interpreting data, and representing and displaying data. Data collecting initiates data management. Data was preserved in well-defined files and directories on a password-protected PC. Before transcribing audiotapes verbatim, they were listened to carefully (where clarifications are needed, an interpreter was approached for assistance). The English translation was verified by native speakers from the health sectors and communities to ensure accuracy. All files were coded, sorted, and password-protected after confirmation to ensure security and simple access.

Statements were grouped into categories/themes that agreed or disagreed with a common concern. After categorizing transcripts and field notes, description and classification began. Common statements were categorized, assigned a code, and related to corresponding theme/s. Then, participants' statements (raw data) were compared to check if they were comparable before interpreting and giving meaning to the themes. Finally, the grouped concepts were tabulated to show important culture sharing themes and categories. This simplifies visualizing and sorting related and contradictory categories with explanations. A descriptive narration discusses important findings and their relationships.

#### Rigor

Rigor in qualitative study refers to the ‘trustworthiness’ or ‘credibility’ of the design, methodology, data collection, description, conclusion, explanation, interpretation, or other form of account. It involves the possibility of obtaining data that fits or corroborates the researcher’s prior notion, belief, or even theories. To obtain rigor in this study, prolonged immersion into the field of study performing persistent participant observation was ensured. With this, understanding and describing what participants say and do in their natural surroundings was ensured. Extensive literature searches were done to understand what the participants say and do. To ensure rich data, sources and procedures were triangulated. Literature review, ethnographic interview, and participant observation were all used to understand the individuals. Until data saturation, informants were continuously recruited and observed.

#### Ethical consideration

Written approval and permission were obtained from the joint Medical Research Council and Gambia Government Research Ethics committee, Research and Publication Committee of the University of the Gambia, Ministry of Health, Officer In-charge (OIC) of the chosen health facility and the consent of participants. Inform consent was obtained from all study participants and/or their legal guardians for those under 18 years of age. All methods were carried out in accordance with relevant guidelines and regulations.

## Results

A total of 22 breastfeeding mothers with infants 4–6 months from KM participated as shown in Table 1. Of the total, nine of the mothers were reported to exclusively breastfed. The mean age of mothers was 28.9 years (SD ± 7.7) ranging from 18 to 43 years. Four in every ten participants were Wollof by ethnicity. Slightly more than half of the participants (54.5%) came from the extended family system. Regarding their educational status, almost half of the mothers, 45.5%, did not have formal education, 18.2%, 22.7%, and 13.6% had primary, secondary and tertiary education, respectively. All the mothers who exclusively breastfeed have a secondary education or higher. Over 75% of women who did not exclusively breastfeed have no formal education. The child's gender and the mother's nursing status also influenced exclusive breastfeeding. The baby weighs 5–10 kg, averaging 6.8 kg (SD ± 1.5). Cereals, tubers, fish powder, milk powders, honey, and warm saline water were used to supplement breast milk.

**Table 1:** Demographic information of the respondents: n = 22, (%)

Characteristics		
Age in years, min. max, mean, (SD)		18, 43, 28.9, (7.7)
Number of co-wives, min. max, (SD)		0, 2, (0.7)
Number of children under 5 years, min. max, (SD)		1, 3, (0.7)
Child’s age in months, min. max, mean, (SD)		4, 6, 5.3, (0.8)
Present weight of child, min. max, mean, (SD)		5, 10, 6.8, (1.5)
Infants recruited in study	Male Female	9 (41) 13 (59)
Infants exclusively breast fed	Male Female	3 (33.3) 6 (46.2)
Ethnic groups’ n (%)	Wollof Mandinka Fulla	9 (40.9) 8 (36.4) 5 (22.7)
Household system n (%)	Extended Family Nuclear Family	12 (54.5) 10 (45.5)
Highest Education attained	None Primary Secondary Tertiary	10 (45.5) 4 (18.2) 5 (22.7) 3 (13.6)
Employment status	Employed Formal Informal Unemployed	3 (13.6) 8 (36.4) 11 (50.0)
Possession of radio/TV	Yes No	19 (86.4) 3 (13.6)
Access to a refrigerator	Yes No	17 (77.3) 5 (22.7)
Weekly hours spent with child	> 100	22 (100)
Common supplementary foods	Cereals: rice, coos, findi and maize powder Tubers: Irish and sweet potato (powder/massed) Milk powders: SMA, lactogen, nurtilac, vitalait Others: dry fish, peanut pap, honey & warm water	

### Breastfeeding settings

Generally, there is no formal setting/environment for breastfeeding in The Gambia. Women breastfeed wherever, whenever, and however they want. They breastfeed at their discretion/need, hence they do it in homes and public areas like markets, hospitals, public transportation, bus stops, stores, streets, etc. Some women can breastfeed while standing in crowded or unhygienic circumstances. In The Gambia, women who breastfeed in public do not need to conceal their breasts. Breastfeeding women traditionally don't wear tight clothing when going out with their infants. In rare circumstances, women who felt embarrassed to breastfeed or prefer to wear tight garments for fashion express their breast milk in a container (typically a tight-fitting basin or bottle with a nipple) to feed their children when going out or leaving them with a caretaker. If they select the latter, a caregiver will feed the child with squeezed/expressed breast milk. High-income homes commonly store expressed milk in a refrigerator, while middle-income households utilize coolers with ice blocks. Poor households either store it in a neighbor's fridge or employ a local cooling system, such as wrapping it in wet cloths, covering it with moist sandy soil, or placing it in jars.

As a custom, new mothers and their babies move to the maternal parent's residence to receive adequate care and support. During this time, family members relieve mothers of all household duties so they can spend time with their babies. Working mothers receives six months of paid maternity leave, whether they work for the government or a private entity. This tradition is strong for two reasons from women’s perspectives: breastfeeding mothers need rest to produce enough breast milk for their baby, especially in infancy and to avoid sexual activity since sperm might contaminate breast milk. Feeding infants sperm-contaminated breast milk causes ill luck, infections, poor growth, and frailty. In the early stage of breastfeeding, only the mother and her family are involved.

### Breastfeeding and family support

I arrived at participant 1 compound at Manjai Kunda around 7am on August 17, 2014. I saw her feeding her baby coos with a basin and spoon in front of her door. She welcomed me and said her child had a fever and vomited last night. Grandma arrived to see why the baby was crying. The baby kept crying despite their efforts. We finally evacuated the baby to the clinics received some medical services. Here are the opinions and response of the maternal mother on giving water to her baby to drink.

*Participant 1:- as many as he wants …anytime …mostly when I drink while carrying him, …oh yes because you will see him look at me. I cannot stand that …I cannot stand my baby staring at me drinking, denying him to drink. This is why I always give him but do not count the number of times. I know it depends on the weather; he virtually drinks more on a hot day than on a cold day.*

### Involvement of males in exclusive breastfeeding

Male involvement remains an important component of comprehensive maternal and child health care. Husbands/partners generally regarded breastfeeding as typical women’s roles and they do not associate themselves with their breastfeeding women. Male’s opinion on exclusive breastfeeding:

*Husband of Participant 1: These breastfeeding issues are more of women activities. Women would know more about it. But for exclusive breastfeeding, my wife is not practicing it. After your interview with her at the health facility, she told me that she discussed this exclusive breastfeeding with you. During that discussion, she made me understand that she has been hearing it at clinics, radios and televisions. Still, because her breast milk is insufficient, she is not practicing it. She also gives supplements with infant formulas such as SMA, celelac, *etc*. I often give her money to buy foods for our baby.*

### Conflict between caring and being cared for

Upon arrival at participant 2 compound at 7:30am, I found her husband in the house who told us that she already left for the market since early morning. Having asked about the baby, he told that madam has gone with the baby- because it was too early; she has to carry the baby on her back to go with her to the market. Shortly before she started laundering, the baby wakes up and she had to stop all the sorting and breastfeed the baby. Having laundered until 12:40 pm, she suspended the laundry and went to the kitchen to start cooking a simple dish called “*mbahal*^1^”. She shuttles between the kitchen and laundry area, a distance of 25 meters. When the baby cries or wakes up, she stops and attends to her. She fed the baby on her lap using a spoon and cerelac mix. After feeding the child, the leftovers were covered and stored in a cupboard in the parlor.

### Cultural food taboos and treatment practices

Dwelling on the cultural and food taboos breastfeeding mothers should avoid. They told me breastfeeding mothers should avoid leafy and green vegetables, watery and hot foods because eating them may give their babies unwanted effects. Another important issue discussed was the reason behind giving babies charm water. In a nutshell, charm water is given to protect infants from evil spirits they said.

*Participant 2: Charm water is usually prepared by the imam. He writes some Quaranic verses on a paper, soak it in water, mixes and filter the water and then put in a bottle. The mother keeps this bottle and will administer it to the child every morning or based on her discretion… but I give it to my child every morning before he eats anything. In the morning, I will use a small teaspoon, put some charm water in, slowly drop it in the child's mouth, and then rub some on his body.*

### Presentation of themes

The data from the interview and observation was analyzed using a constant comparison method. Manually transcribed audio data was coded with observation notes. Having coded the data; codes with shared characteristics were categorized. Maximizing variation and developing linkages further saturated the resulting categories [34]*.* Table 2 shows six themes, each with subthemes, related to lactating women's cultural beliefs, attitudes, and perceptions of exclusive breastfeeding in The Gambia.

**Table 2:** Summary of themes and sub-themes

Themes	Sub-themes	Response summary
Mothers’ food taboos for infants’ wellbeing	*Eating green/leafy vegetables or liquid foods causes infants diarrhea*	*I stopped eating green and leafy vegetable since I delivered because my mother told me that it may cause diarrhea to my child (Id7p43l6-7)*
	*Eating hot food causes infant fever*	*I don’t want my child to develop fever that is why I stopped eating all these hot foods like; pepper, ginger and black pepper despite I like them very well (Id14p88l15-16)*
	*Eating snail makes infants salivate a lot*	*Eating snails during breastfeeding makes infants salivate a lot (Id9p55l13)*
Cultural interpretation of the physical effects of breastfeeding	*Weight loss is inevitable during breastfeeding*	*Weight loss is inevitable in breastfeeding because babies suck all the nutrients mothers eat (Id5p33l9)*
	*Excessive sucking results to nipple inflammation*	*my nipples are inflamed because this child (pointing at her child) breastfeeds a lot (Id11p67l5-7)*
	*Night breastfeeding causes morning headache*	*I avoid night breastfeeding because it gives me morning headache anytime I do (Id8p46l2-5)*
	*Sitting for long time breastfeeding hurts back*	*…this is usual for me. I started observing it long time ago. Anytime I sit for long breastfeeding, I do encounter backache (Id8p49l19-21)*
Drinking water being indispensable for infant survival	*Infants cannot survive without drinking water*	*Oh no! Breast milk alone cannot satisfy infants taste. They also need to drink after breastfeeding to quench their taste. Not drinking water may jeopardize their life you know (Id1p9l5-7)*
	*Weather is too hot to deny infants water*	*Infants cannot survive without drinking water in this hot weather… it is very hot you know! Even adults will die if they don’t drink water in this hot weather more so infants (Id16p94l7-8)*
Instilling Islamic faith and shielding infants against evil spirits	*Ingesting charm water immediately after birth make infants pious Muslim*	*I cannot exclusively breast feed because as a Muslim, the first thing I would like to give to my baby is charm water… this is what I found and this is what I have been doing (Id1p1l2-4)*
	*Drinking charm water protects infants against evil sprit*	*Infants are very prone to evil spirits that is why we give charm water regularly to protect them against evil spirits. (Id3p18l11-12)*
	*Drinking water softens babies heart and makes them calm and soft-hearted*	*Not drinking water causes hard-heartedness making infants merciless as they grow to adults (Id2p12l22-23)*
Basing milk adequacy on breast size	*Small breast size does not produce adequate breast milk*	*I wish my breast were big…em em I really envy mothers with big breast because they do have adequate breast milk to breast feed (Id14p89l17)*
	*Big breast size produces adequate breast milk*	*I do produce adequate breast milk because I have big breast (Id8p51l2-7)*
Nurturing men’s physical strength early at infancy	*Starting supplementary foods early for baby boys makes them physically strong adults*	*I want my boy to be a strong man that is why I’m giving him food early. breast milk alone is very light for baby boys (Id18p103l7)*

#### Theme 1: mothers’ food taboos for infants’ wellbeing

Baby’s welfare is traditionally based on the types of food mothers eat during breastfeeding. Most of the mothers believed that eating certain foods during breastfeeding influences their baby's health. For this reason, most mothers say they either stopped or minimized eating some foods during breastfeeding.

##### Eating green/leafy vegetables or liquid foods causes infants diarrhea

Eating green/leafy vegetables and liquid foods was associated with infants’ diarrhea. As reported by a mother:

*Respondent: I stopped eating green and leafy vegetable since I delivered because my mother told me that it may cause diarrhea to my child (Id7p43l6-7).*

By referring to green/leafy vegetables she means leafy vegetables that are green in color; resembling the stool of infants with diarrhea. Such foods include sorrel, lettuce, cabbage, cassava and potato leaves. Haven enquired the reason of prohibiting breastfeeding mother from eating green leafy/liquid foods, I was told that:

*Respondent: Eating such foods may contaminate mothers breast milk… and when the child sucks that milk, the color of his/her stool changes to green and continuous feeding on such milk may cause diarrhea (Id7p43l8-11).*

On the other hand, the respondents reported eating liquid foods such as porridge, pap, “ebbeh” and “gari” as food taboos. However, it should be noted that there are controversies about eating such foods. While on one hand some believe that it may boost milk production; others on the other hand reported that eating these foods increases the frequency of defecation among infants; thus, leading to diarrhea. As she reported, most other mothers believed that liquid foods do not have adequate nutrients to sustain both mothers and infants, and the excess water content causes infant diarrhea. The deep-rootedness of these beliefs has been well noticed during the participant-researcher interactions. Participants discreetly nodded in low and soft voices that a breastfeeding mother should eat these foods under no circumstances. They repeatedly express that those foods are good for the mother and her baby. Foods like “*mbaha*” and “*domoda*” were mentioned by many as good foods to boost milk production without causing undesirable effects on infants.

##### Eating hot food causes infant fever

The mothers also believed that “*eating hot foods during breastfeeding may cause infant fever”.* Due to their natural sharp taste, mothers believe that these foods are potential causes of infant fever. In a conversation, a mother reported that:

*Respondent: I don’t want my child to develop fever that is why I stopped eating all these hot foods like; pepper, ginger and black pepper despite I like them very well (Id14p88l15-16).*

Hot food in their context denotes spicy foods such as pepper, ginger, onion, and black pepper. To some, it may even be the cause of fever in adults.

Hot foods as reported, may be a potential cause of infant fever because of the belief that it may contaminate the breast milk. For that reason, it is forbidden to consume such foods during breastfeeding.

##### Eating snail makes infants salivate a lot

In addition to diarrhea and fever, the data also showed mothers associating the “*eating of snails to infants’ salivation”.* Because dribbling is a common nature of the mentally retard or imbalance people in the community, mothers do show a sense of fear of seeing their babies hyper drooling. People will see their baby be mentally imbalanced. Due to the stigma, nobody wants to be blamed or nurse a dribbling baby, breastfeeding mothers do not eat snails at all despite it’s a common food women like so much.

#### Theme 2: cultural interpretation of the physical effects of breastfeeding

Data from the participants also revealed several cultural perceptions and interpretations of the physical effects of breastfeeding. While the effects are the same across the mothers, there is a variation in the interpretation of the causes of those physical effects; others put them on a normal footing and consider them inevitable as far as breastfeeding is concerned.

##### Weight loss is inevitable during breastfeeding

The data revealed an established belief that breastfeeding mothers must lose some weight- no matter what; because the breastfeeding baby sucks the nutrient she eats. Though reported as one of the most abhorrent encounters breastfeeding mothers have to live with, it was not a major deterrent to breastfeeding. Because of the belief that baby’s suck all the nutrients’ mothers eat during breastfeeding, it is commonly perceived that losing weight during breastfeeding is an inevitable norm. However, because putting on weight is a sign of enjoyment and living large among Gambian women, most participants reported that they don’t like losing weight. For this reason, some would supplement their breast milk to minimize breastfeeding frequency. A participant said this during a conversation:

*Respondent:* …*my baby sucks a lot. That is why I am losing weight very fast; In fact, at one point I was ashamed of going out simply because I was losing my real woman shape. I felt the way people stared at me in the neighborhood…they feel I was not living well. But since I started giving him cerelac and coos ogi, his breastfeeding has reduced a lot and thanks to God I am putting up some weight now (Id11p72l13-17).*

The above statement is a typical demonstration of how Gambian women value and compete for weight gain; for them, a real woman should have enough flesh on her frame. In respect to this, losing weight is something they can’t afford to put up with. Some would rather give up breastfeeding or even take supplements to beef up their shape and look like real women. In addition to the belief that infants suck mothers’ nutrients, causing weight loss, inadequate sleeping was also claimed to be a contributor to weight loss during breastfeeding.

*Respondent: My weight loss is due to inadequate sleep… I don’t have enough sleep both day and night. I can only rest when this child sleeps (Id8p49l17-18).*

##### Excessive sucking results in nipple inflammation

Nipple inflammation, which many reported was a consequence of excessive sucking, was another challenge that breastfeeding mothers encountered. While a mild inflammation was bearable and considered normal by the mothers, the soreness was a detested encounter that influenced the mother's breastfeeding practices:

*Respondent: nipple inflammation is one of the things I hate most in breastfeeding… it is my biggest problem (Id11p67l1-2).*

##### Night breastfeeding causes morning headache

Mothers also reported morning headaches as another challenge they encounter during breastfeeding. A mother in respect to this said that:

*Respondent: I try by all means to avoid night breastfeeding because anytime I do it, I wake up with headache in the morning. As a result, I use to feed the child with cerelac or coos ogi before going to bed… once the child is full, she sleeps the whole night with few sucking (Id8p46l2-5).*

Though the physiology of how night breastfeeding causes morning headache was not clearly explained, most attributed it to sleep interruptions and inadequate sleep.

*Respondents: … My morning headache is due to improper sleep at night. The baby keeps interrupting my sleep… he sucks a lot at night; waking me up almost every hour to breastfeed. Because of that I cannot open my eyes in the morning… I will get up sleepy with headaches in the morning (Id12p77l18-21).*

##### Sitting for long-time breastfeeding hurts back

Backache, attributed to sitting for long breastfeeding, emerged as a major encounter among the mothers. Mothers reported that their back hurts when they sit for long breastfeeding. A mother says anytime she wants to breastfeed for long she has to lie down because from her experience, she suffers backache whenever she sits for long.

*Respondent: … this is usual for me. I started observing it long time ago. Anytime I sit for long breastfeeding I do encounter backache (Id8p49l19-21).*

As a results of the pain associated with sitting and breastfeeding, some of the mothers reported that they usually lie down while breastfeeding.

*Respondent:… unless otherwise, I don’t usually sit down while breastfeeding. This is because my back hurts me anytime I sat for long breastfeeding (Id5p33l3-4).*

#### Theme 3: drinking water being indispensable for infants’ survival

##### Infants cannot survive without drinking water

As a custom in many other parts of the world, mothers in the Gambia also have the stronghold that drinking water is necessary for infants’ survival. Though some mothers show signs of approval and commitment to exclusive breastfeeding, the value attached to giving infants water is a major barrier to the practice. To most, water is necessary for life, and infant survival is impossible without drinking water. The following dialogues epitomize that:

*Respondent: Hmmm! All I know is I will never allow my child to be thirsty. I always give him to drink; mostly when I drink. Yes! Because infants are also human beings, they need to drink like any other living thing to survive (Id3p22l7-9).*

Mothers also believed that denying babies water at infancy may make them reject water later in life. And considering the value attached to drinking water, rejecting water is considered as a risk for human existence. Though some mothers do show some commitment to breastfeeding their infants exclusively, most are unsure whether denying their infants’ water will jeopardize their life; thus, their love for their babies does not allow them to take any risks. As added by another mother.

*Respondent:… because I heard infants risk dying when denied water… I… oh yea I don’t want to be a victim… I don’t want my baby die (uh!); that is why I’m giving my baby water. I love my child so much and cannot risk his life (Id8p48l5-7).*

##### Weather is too hot to deny infants’ water

The hot weather was another issue mothers expounded on, justifying the necessity of drinking water for infants’ survival. A woman in a conversation said that:

*Respondent: infants cannot survive without drinking water in this hot weather… it is very hot you know! Even adults will die if they don’t drink water in this hot weather more so infants (Id16p94l7-8)*.

In dwelling on the importance of water, she said:

*Respondent: Why should I deny my baby water when I saw nurses mounting water for babies when they are admitted at the health center? Irrespective of the baby’s age, the first thing nurses will do is mount water on admission… if you see nurses give water; even to babies, it is clear that babies cannot survive without water (Id2p17l3-6).*

To her, if infants under six months should not have been given water, why should nurses mount water for infants under six months; a question she posed to me. But we laughed about it and the discussion continued.

#### Theme 4: instilling Islamic faith and shielding infants against evil spirits

##### Ingesting charm water immediately after birth make infants pious Muslim

Islam is the major religion of the Gambia, with around 90% of the population being Muslims. As the religion dictates, parents must instill the Islamic faith in their young ones. First, women pledge their newborns to Allah and choose to raise them with the Islamic faith and fear of Allah. Every mother wants her child to grow up with the Quran and Sunnah's moral principles. Mothers use the Holy Quran as a guide to raise their children so they may know the Lord and have a heart filled with love and Allah's love. Infants whose first food is the Holy Quran are believed to grow up to be pious Muslims.

Charm water is spiritual water created of Quranic verses written on A4-sized white papers, soaked in water, mixed, and put in bottles for infants to swig with the index or middle finger. In modern times, bottles with nipples or teaspoons are utilized. Though anyone can give it, grandparents usually do. As reported, its collection and administration delayed breastfeeding following delivery. Participants in similar situations reported waiting for the imam's holly water before providing anything to their babies.

*Respondent: When I delivered, the imam was immediately contacted for charm water… before giving anything to the child; I used the charming water to wash my breast and then gave the child to drink before breastfeeding him. With this, my baby will grow with the faith and fear of Allah, behave in line with moral values and have a powerful mind and character… mashallah! … (smiles) what a better gift can a mother bestow than this (Id6p39l6-11).*

The strong belief that infants must be raised with such powerful sincerity and virtue has resulted to mothers maintaining the first thing to be ingested by infants to be charm water.

##### Drinking charm water protects infants against evil spirit

Along with the religious responsibility, children are protected from bad spirits. Infants are prone to bad spirits, according to tradition. Mothers seek charm water from astrologers, marabouts, and imams to protect their children from bad spirits. The following responses were noted concerning that:

*Respondents: Infants are prone to evil spirits; we give them charm water regularly to protect them against evil spirits. (Id3p18l11-12).*

Her statement can be linked to the perception of mothers associating most of the common childhood illnesses, misfortunes and discomforts to the curse of evil spirits. Like a mother with a history of sleepless children reported:

*Respondent: When my child was born, he does not sleep at night… he cries the whole night but since I started giving the charming water that has faded now. I know the benefit of charm water; I always give it to my baby (Id5p32l3-5).*

##### Drinking water softens baby's heart and makes them calm and soft-hearted

Since being a Muslim translates to being modest and sympathetic, mothers further stated that:

*Respondent: not drinking water causes hard-heartedness making infants merciless as they grow to adults (Id2p12l22-23).*

Mothers have frequently emphasized water's capacity to soothe the heart and make one compassionate. Denying newborns water is considered harsh by some. Others question how a mother can drink and deny her children. The statements below are excerpts of some mothers’ responses in respect to that:

*Respondent:* … d*rinking water is very good because it softens the human heart. Just see how short-tempered you will be when thirsty…this is why water is taken to cool one’s temper when angry (Id8p48l6-8).*

Most, if not all, cultures provide angry people water to cool off. Since mothers want to nurture sympathetic children, they always offer them water.

#### Theme 5: basing milk adequacy on breast size

##### Small breast size does not produce adequate breast milk

The data further showed that breast milk adequacy is based on breast size. Small-breasted mothers believed they could not produce sufficient breast milk to meet their babies' demands. For this reason, they were more likely to supplement their breast milk with infant feeds to meet their baby’s dietary requirements. Not only do the community members, but mothers themselves also pay great attention to their breast size. Those with small breasts are always with the notion that their small breast cannot produce adequate breast milk for their babies, for this reason, they need to supplement the breast milk to feed their infants adequately. The strong association between milk adequacy and breast size could be sensed from her statement. Most mothers are worried about their breast size and would quickly conclude their diminished or scanty milk production is due to their small tit size. Due to the above reasons, being breasted is considered a pride among breastfeeding mothers, and small-breasted mothers were heard to be envious of breasted mothers. As mentioned in a dialogue:

Respondent: *I wish my breast were big…em em I envy mothers with big breasts because they have adequate breast milk to breastfeed (Id14p89l17).*

Due to the misconception that small breasts do not produce enough milk, mothers with small breasts are typically discouraged from breastfeeding, resulting in greater supplementation. Big boobs are always preferred, and they envy breastfeeding mothers. Due to their tits, affected mothers do not breastfeed exclusively.

##### Big breast size produces adequate breast milk

I also asked breastfed mothers about their large breasts. They are more motivated to breastfeed as they have more milk. A breasted mother says she has plenty breast milk because she has large breasts. In her statement, she said*:*

*Respondent: Oh yes, I produce adequate breast milk because I have big breasts… while laughing, she cheerfully said that my baby could not finish my breast milk… laughs…she added that my breast would still be full… I can feel the heaviness even after breastfeeding him to his satisfaction. Anytime she misses breastfeeding for few hours, I encounter engorged breasts. Sometimes the engorgement is so tense that I must express the breast milk to relieve myself of the pain (Id8p51l2-7).*

#### Theme 6: nurturing men’s physical strength early in infancy

##### Early initiation of starting supplementary foods for baby boys

Another barrier to the practice of exclusive breastfeeding was the belief that starting supplementary foods early for baby boys is the beginning of lifelong eating habits that can contribute to their overall physical strength as they grow into adulthood. This was seen as a highly respected perception among the studied women. In many statements, women strongly believe feeding baby boys with breast milk alone will compromise their physical strength when they grow. By referring to *breast milk is very light*, she means that breast milk alone cannot satisfy baby boys; thus, the need to supplement it with supplementary foods. It is a common belief that breast milk cannot meet the nutritional requirements of baby boys and that feeding them exclusively on breast milk will compromise their physical strength. Due to this well-grounded concept, breast milk is usually supplemented early for baby boys.

Due to the belief that men are and should be physically stronger to shoulder the day-to-day activities; to earn income to feed and shelter their families, mothers take the responsibility to nurture and develop the physical strength of their sons early in infancy. To nurture this strength, mothers believe that breast milk alone is insufficient to nurture men’s physical strength. As heard from a respondent that:

*Respondent: You cannot compare men with women… even as adults’ men eat more than women…men are stronger than women and for that reason, they should also start eating (supplementary foods) early at infancy (Id8p46l12-14).*

Such belief could also be attributed to the patriarchal nature and stereotypical idea of the Gambian people that men should appear stronger, substantial and vigorous all the time more than women or, if not, be considered social freaks. In respect to this deep-rooted culture, male children are introduced to supplementary foods early to develop their physical strength.

## Discussion

Infant feeding practices in the Gambia cannot be separated from the culturally embedded context and meanings. This study has highlighted the existing cultural beliefs, attitudes and perceptions of the mothers; thus, revealing the experiences and barriers to the practice of exclusive breastfeeding.

### Mothers’ food taboos for infants’ wellbeing

Cultural beliefs and attitudes affect breastfeeding success. Food beliefs and perceptions affect how mothers breastfeed. This study indicated that Gambian breastfeeding mothers have various food taboos. Since birth, research participants avoided one or two foods that could affect their offspring. Similar findings were reported elsewhere in India; breastfeeding parents omitted particular foods from their diets to prevent or treat infant or self-health issues. Indian mothers avoided green leafy and fibrous vegetables during breastfeeding for fear of harming babies or themselves [35, 36].

Green vegetables and watery diets may increase newborn diarrhea risk. As a result, mothers avoided these foods. In Nigeria, breastfeeding mothers avoided plantains and bananas because they believed they contaminated breast milk and caused newborn diarrhea. The study also found that women believe snails induce newborn hypersalivation. According to a Nigerian study, mothers forbid okra because they believe it makes babies slobber excessively [37]. In their study of dietary taboos for breastfeeding mothers, Mexican women devoured hot foods and prohibited cold items [38]. The inconsistency may derive from Gambian beliefs that coolness comes from water and heat from the sun. They also considered the cultural concept that water makes one warmhearted, which might be attributed to the ancient custom of encouraging the consumption of cold foods (fruits, nuts, corns, beans, etc.), especially by women-to-be and new mothers. In a study among Chinese mothers in Ireland claimed to consider cold foods hazardous to their kids, contradicting this study's findings [39].

### Cultural interpretation of the physical effects of breastfeeding

Breastfeeding is the most accepted baby feeding approach because it is cheaper. Successful practice requires mothers to endure physical implications. Different parts of the world view breastfeeding's physical consequences as barriers, according to studies. Some mothers do not breastfeed because of weight loss [40]. Understanding the physical impacts can help with prevention and treatment. This study showed cultural interpretations of mothers' side effects. Some are normal, but others hinder women's breastfeeding. Breastfeeding infants were considered to cause weight loss by depleting their mother's energy and nutrients. Rural Gambia mothers blamed excessive breastfeeding for weight loss and anemia [20]. Due to the perceived benefits of breastfeeding as the most popular infant feeding approach, Gambian mothers do not discontinue breastfeeding due to weight loss and fatigue from frequent breastfeeding [21].

Consistent with a study in Zambia revealed that sore nipples as one of the major barriers to breastfeeding among mothers [41]. In this study, a mild nipple inflammation or discomfort was considered normal, but a too sore or painful nipple, which was said to be produced by excessive sucking, was found to be a deterrent to breastfeeding since the barrier (sore and painful nipple) outweighed the perceived benefits. In earlier and current studies, mothers linked nipple difficulties to poor latching and placement [2, 22, 40]; contradicting the findings of this study. Backache was another reported difficulty with breastfeeding. Breastfeeding causes backache for some mothers. Despite the backache, mothers choose this as an alternative and they have historically associated breastfeeding with pain and weariness [42].

### Drinking water being indispensable for infants’ survival

Mothers around the world have cited water's importance for newborn survival. Breastfeeding is largely considered as the best infant feeding option in Gambia, as evidenced by the high practice at 99% and duration rates at 24 months [31]. Exclusive breastfeeding was difficult because women believe infants will die without water. The women say babies can't survive without water. Mothers suggested babies should drink water. Due to the value of drinking water and the fear of newborns rejecting water, survey participants felt no guilt giving infants water. Even though this research was conducted in urban Gambia, rural mothers underline the importance of water [20, 22].

Noting how infants lose water through sweating and urination, mothers believe that only uncaring and barbarous mothers would deny their infants' water. Studies found that solely breast milk is sufficient for healthy infants (less than six months) and that no water supplementation is essential even in arid and hot climates [43, 44]. In this study, women who exclusively breastfeed their infants are disparaged since they deprive them of water. A mother who solely breastfed her child revealed to the researcher that her relatives thought she was neglecting to give the child water. In Ghana, mothers argue that infants need additional water to quench their thirst because they feel that depriving them of it will harm their health [45]. Early water introduction improves child survival and development, contradicting WHO/UNICEF recommendations [1, 46].

### Instilling Islamic faith and shielding infants against evil spirits

As a religious directive in Islam, new mothers are recommended to breastfeed their infants for two years. Through thick and thin, fathers are also commanded to support their wife’s in any circumstances that may affect breastfeeding [47]. The advantages of practicing good Islam outweigh those of exclusive breastfeeding in the Gambia. The Quran is used by parents to raise their children in the Islamic nation of Gambia. This is a result of the clergy's ongoing "dahwa" urging Muslims to abide by Allah's commandments, particularly with regard to how they rear their children. Parents are advised that it is their responsibility to teach their children the Islamic faith and that their lord would ask them over how they raised them. A theory that may have an impact on exclusive breastfeeding Holy water is frequently given to newborns by mothers, who hope that doing so will help them grow up to be devout Muslims. A custom that was inspired by the Prophet Muhammad (SAW), who would offer prayers and ceremonies to a newborn [47]. The ritual known as “*Tahneek”* is practiced with hopes that it will make infants good and obedient servants of God.

It is said that water may soothe the heart and subdue anger. The perceived advantages of providing water to infants outweigh the significance of withholding it, as withholding water is said to result in hardness of heart, turning infants into temperamental, obstinate, harsh, and unforgiving adults, which is against appropriate Muslim behavior. Mothers persisted on giving their infants water because they wanted to create obedient, God-fearing children. Many mothers claimed that water softens the heart and develops warm-hearted adults in babies. This idea might have originated from the Gambian practice of giving angry people water to make them calm down.

Many underdeveloped countries, especially Africa, believe newborns are susceptible to bad spirits and employ charm water to protect them. In SSA, women give children herbal water to protect them from bad spirits or alleviate their curses [48]. In this study, mothers reported giving newborns holy water to ward off bad spirits. Kenyan women reportedly use holy waters/traditional plants to treat sick infants or guard them from evil [49]. This conclusion contradicts research that found Zambian mothers favored exclusive breastfeeding and opposed giving the baby anything before breastfeeding. Zambians are more familiar with exclusive breastfeeding than their neighbors [41].

### Mothers’ opinions regarding milk adequacy based on breast size

Understanding breast anatomy, lactation physiology, and good breastfeeding technique are necessary for successful breastfeeding. Women must be aware of what affects milk production in order to sustain it. Although Asian pregnant women have smaller breasts than Western pregnant women, Pharuhas, C., has noticed an increase in concern over maternal breast size and milk production in Asian pregnant women [50]. This study found that optimal milk production was significantly influenced by breast size, and that breastfeeding exclusively was difficult for women with small breasts. Conversations found that breastfeeding women blamed breast size for having enough milk. A mother wishes she had large breasts so she could properly breastfeed her child. According to research, there is no correlation between breast size and milk production since the former depends on infant demand rather than breast size [51].

### Nurturing men’s physical strength early at infancy

In patriarchal countries like the Gambia, where men remain responsible for feeding, housing, and caring for their family, men's ability to do physical labor is crucial to their well-being and independence. Workability is crucial. Nutrition (especially in infancy) is a determinant in older age physical strength. Men are physically stronger than women and should start taking supplements early. Rural Gambian mothers believed breastfeeding boosted infants' strength, according to Janneh et al. [22]. Similar statements are repeated. Parents gave their newborns additional nutrients since they believe breast milk alone will hinder their physical strength and growth for six months. Start taking vitamins early to improve strength. Postnatal diet may influence muscle mass and strength forever [37]. Elsewhere in a neighboring country, mothers in Senegal have been reported to associate breastfeeding with improved growth in length but not strength [52]. However, this disagrees with Siân M.R. et al. 2012 whose study concludes that greater exposure to breast milk in infancy is associated with greater strength in men [53].

### Study limitations and strengths

A possible bias in this study could be linked to the researchers' recruitment procedure and health background. Nurses, who offered the same women's health service, were used to recruit participants for the study. These may have made it difficult for the mother to decline to participate in the study. Furthermore, a social desirability bias might be introduced where the mothers answered or behaved in manner to satisfy the researcher. The study was conducted in urban municipality with a multicultural and diverse population where almost half of the Gambian population resides. Thus, the results will likely represent the existing cultural beliefs, attitudes and perceptions of breastfeeding mothers in the Gambia.

## Conclusion

The study found that promoting exclusive breastfeeding without knowing mothers' sociocultural ideas and views was nearly difficult. Family members, especially grandparents, have a significant role in infant feeding. Since new mothers are relocated to their parental households following delivery, maternal grandmothers are the most influential during the first few months after childbirth. Indoctrinating children in Islam and protecting them from bad spirits hampered exclusive breastfeeding (as mothers insisted on giving charm water immediately after birth before initiating breastfeeding). Small breasts inhibit breastfeeding practices among mothers. Large-breasted women breastfed more often. The study found no link between breast volume and early milk output. Feeding baby boys extra nutrients early can build their bodies. More research is needed to identify ways to influence lactating women's cultural beliefs, attitudes, and perceptions to favor exclusive breastfeeding. The origin and social context of breastfeeding practices need more exploration in both urban and rural settings. The study findings could benefit healthcare providers in informing policies and designing culturally adaptive and acceptable community-based breastfeeding intervention programs in resource-limited settings.

## Data Availability

Data for this study could be available upon request from the corresponding author.

## Footnotes

1 *A common Gambian cuisine; prepared with rice, peanut powder and dried fish.*
